# Clinical features of patients with non-metastatic lung cancer in primary care: a case-control study

**DOI:** 10.3399/bjgpopen18X101397

**Published:** 2018-04-07

**Authors:** Marcela Ewing, Peter Naredi, Chenyang Zhang, Lars Lindsköld, Jörgen Månsson

**Affiliations:** 1 PhD student, Department of Public Health and Community Medicine/Primary Health Care, Institute of Medicine, Sahlgrenska Academy, University of Gothenburg, Gothenburg, Sweden; 2 Professor, Department of Surgery, Institute of Clinical Sciences, Sahlgrenska Academy, University of Gothenburg, Sahlgrenska University Hospital, Gothenburg, Sweden; 3 Statistician, Regional Cancer Centre West, Sahlgrenska University Hospital, Gothenburg, Sweden; 4 Senior Lecturer, Department of Applied Information Technology, University of Gothenburg, Gothenburg, Sweden; 5 Professor, Department of Public Health and Community Medicine/Primary Health Care, Institute of Medicine, Sahlgrenska Academy, University of Gothenburg, Gothenburg, Sweden

**Keywords:** diagnosis, general practice, lung cancer, non-metastatic, primary health care, Sweden

## Abstract

**Background:**

Lung cancer (LC) kills more people than any other cancer globally, mainly due to the late stage of diagnosis.

**Aim:**

To identify and quantify the prediagnostic features of non-metastatic lung cancer (nMLC) and to compare the clinical features in GPs’ chest X-ray referral letters with the clinical features (expressed as diagnostic codes) in medical records.

**Design & setting:**

A population-based case-control study was conducted using diagnostic codes from national and regional healthcare databases in Sweden.

**Method:**

In total, 373 patients diagnosed with LC in 2011 (of which 132 had nMLC) and 1472 controls were selected from the Swedish Cancer Register (SCR) and regional healthcare database, respectively. Diagnostic codes registered in medical records from primary care consultations in the year before LC diagnosis were collected from the regional healthcare database. Odds ratios (OR) were calculated for variables associated with nMLC. The GPs’ referral letters for chest X- ray were retrieved from the regional repository for radiology.

**Results:**

Clinical features with the highest OR were vitamin B12 deficiency anaemia (OR 6.7, 95% confidence interval [CI] = 1.6 to 27.9), dyspnoea (OR 5.0, 95% CI = 2.0 to 12.7), and chronic bronchitis (OR 5.0, 95% CI = 1.3 to 18.6). Clinical features that were GPs’ reasons for requesting chest X-ray were almost three times more frequent in referral letters compared to the corresponding diagnostic codes in the medical records.

**Conclusion:**

Patients with nMLC could not be identified by symptoms. The clinical features in referral letters for X-ray were more frequent than corresponding diagnostic codes from medical records.

## How this fits in

Late-stage diagnosis is a main reason for the high mortality of LC. Different risk assessment tools have been developed for GPs in order to detect LC earlier by clinical features. This study shows that patients with nMLC could not be identified by clinical features. However, despite the lack of this specific knowledge, GPs’ referrals for a chest X-ray resulted in a 40% detection rate of nMLC.

## Introduction

LC is one of the deadliest and most common cancers in the world. With an estimated 1.8 million new cases in the world each year, this cancer is responsible for almost one cancer death in five.^1^ LC is the fourth most common cancer in Europe with >410 000 new cases diagnosed in 2012.^1^ The high mortality is due to both late-stage diagnosis and delay in treatment.^2–5^ In the UK, 46 000 new cases of LC were diagnosed in 2014, and half of the patients with known stage were diagnosed at Stage IV (metastatic disease).^6^ In Sweden, 4194 patients were diagnosed with LC in 2015, and 3626 died from it.^7,8^ Despite having high survival rates for many types of cancer, Sweden has poor survival rates for LC.^9^ The relative 5-year survival rate for LC in Sweden is 18%.^10^ The low survival rate is mainly due to late-stage diagnosis. More than 50% of all Swedish patients with LC are diagnosed at Stage IV, with a relative 5-year survival rate of 2.6%. However, when LC is diagnosed at Stage I, the relative 5-year survival rate is 63.8%.^10^ In order to increase survival rates for patients with LC, the most important factor is being able to identify those with a potentially curable disease. There is value in identifying patients at Stage I–III, whose LC has yet not spread, because they, as a group, have a relative 5-year survival of 36.1% versus 2.6% for Stage IV cancer.^10^

Screening of target groups has been discussed as a method for early diagnosis of LC. Low-dose computed tomography (LDCT) in a defined population of high-risk persons has shown high sensitivity and acceptable specificity.^11^ Publications from different LC screening trials show that up to 70% of screen-detected, non-small cell LCs were found in Stage I, compared to around 15% found in routine clinical care.^12^ LDCT is currently being used as screening for LC in the US.^12^

GPs are important in cancer diagnostics because in countries like Sweden, Norway, Denmark, and France, approximately 70–87% of patients with cancer are diagnosed in a primary care setting.^5,13–15^ Because Sweden possesses unique total population-based databases, a case-control study could be conducted using regional databases for healthcare and diagnostic imaging in combination with the national cancer register.

This study aimed to:

- identify the clinical features of nMLC in primary care before the diagnosis is made; and
- validate the clinical features from the regional healthcare database with clinical features in GPs’ referral letters for chest X-rays.

## Method

### Study design

A total population-based, case-control study was designed, using the SCR and a regional healthcare database in Region Västra Götaland (RVG), Sweden. This region, which has 1.6 million inhabitants, is situated in the south-west of the country.

The SCR, which was established in 1958, is one of the oldest disease registers in the world and has high validity.^16^ All physicians, including pathologists, in Sweden are obliged by law to report all incident cases of cancer in both living and deceased patients to the SCR.^17^ Each patient has a unique personal identity number, which all Swedish residents acquire either at birth or when they immigrate to Sweden.

The regional healthcare database was established in RVG in 2000. It covers all hospitals, specialised outpatient care centres, and all private and public primary healthcare centres. The database includes a place of residence, age, sex, healthcare contacts, and diagnostic codes for diagnoses and surgical procedures.^18^ Physicians are obliged to enter codes for a patient’s current diseases or symptoms into the patient’s medical records at each consultation. The reimbursement system for primary care providers is based partly on the disease burden of the patients, which is identified by diagnostic codes reported to this database. The diagnostic codes are usually expressed in International Statistical Classification of Diseases and Related Health Problems, 10th revision (ICD-10) classification,^19^ but the International Classification of Primary Care (ICPC-2)^20^ is often used in primary care for its better descriptions of symptoms.

In 2006, the Enterprise Information Archive (EIA), a regional database for radiology information, was established. It allows both textual information and images to be shared (stored and distributed) from every radiology department in the RVG.^21^ Both publicly and privately-financed radiology clinics send information to this database.

### Study population

All patients in the RVG with LC diagnosed in 2011 were identified from the SCR. As this study was total population-based, no sample size was calculated.

Patients and matched controls were investigated for primary care diagnostic profiles. The inclusion criteria were:

- being diagnosed in RVG with LC;
- being alive at the time of the cancer diagnosis;
- being aged ≥18 years; and
- having visited the GP during the year before cancer diagnosis.

Individuals were excluded from participation if they:

- lacked controls;
- had a previous cancer diagnosis in the SCR (1991–2010); or
- had a metastasised, Stage IV LC.

Patients with a previous cancer diagnosis registered in the SCR during the 20-year period before 2011 were deliberately omitted, to avoid consultations in primary care being a control or related to a previous cancer. The controls were selected from the regional healthcare database. They had the same inclusion criteria as the patients with cancer, with the exception of a cancer diagnosis. Only controls from RVG who had visited a GP in primary care between 1 January 2010 and 31 December 2011 were eligible. Four controls were matched to each case for age, sex, and primary care unit.

### Data collection and study measurements

The unique personal identity numbers of both cases and controls were linked to the regional healthcare database. All the data concerning diagnoses and dates of consultations with a GP between 1 January 2010 and 31 December 2011 were collected. The data extracted included diagnostic codes according to the Swedish version of the ICD-10;^22^ or the Classification of Diseases and Health Problems 1997 Primary Care (KSH97-P). This is an abbreviated version of ICD-10, adapted to Swedish primary care to facilitate diagnostic coding.^23,24^

The unique personal identity numbers of cases were linked to the EIA database. GPs’ referral letters for chest X-ray — containing detailed clinical information with risk factors, symptoms, and signs from physical examinations and pathological laboratory results — were retrieved either from the EIA database or other repositories.

Two medical oncologists and a GP, independently of each other, coded the clinical features in all the referral letters for chest X-ray, using the ICPC-2 codes because these are more symptom-based. Where the codes were not consistent between the three coders, a consensus was reached on the final coding. These codes were then compared with the ICD-10 diagnostic codes from medical records in the healthcare database. As the authors only had access to diagnostic codes, the referral letters provided the reasons for chest X-ray referrals. In addition, because a more symptom-based coding classification was used (ICP-2), a comparison was made between how well the clinical features in referral letters corresponded to the clinical features coded in a less symptom-based classification (ICD-10) in the regional healthcare database.

### Diagnostic codes

All the diagnostic codes registered when patients with cancer and their controls consulted their GP during the year preceding their cancer diagnosis were studied. Because >6000 different diagnostic codes were received for patients with nMLC, the number was reduced by merging the four-character diagnostic codes to the closest three-character diagnostic codes, according to clinical relevance.^15^ Finally, 575 codes remained that had occurred in ≥1% of either cases or controls.

### Data analyses

The 575 diagnostic codes were used as variables for univariable conditional logistic regression. Those found to be associated with cancer entered multivariable analyses, after which a list of statistically significant variables associated with LC was compiled. All analyses were performed using the statistical software R (version 3.0.1).

## Results

### Cases and controls

**Figure 1.**
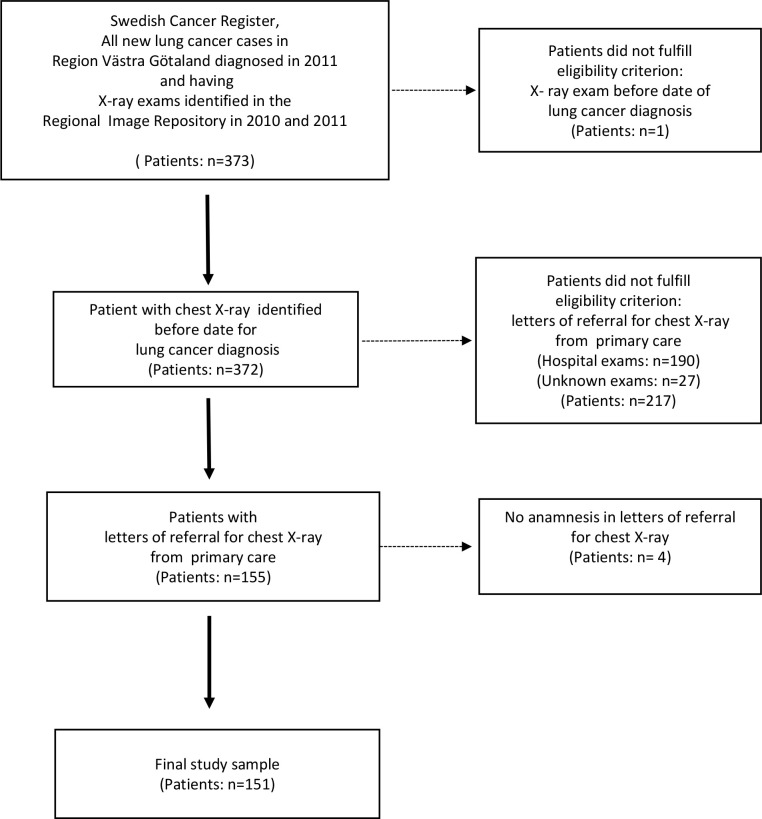
Selection process of patients with lung cancer in primary care with first referral to chest X-ray examination from primary care.

In total, 373 patients with LC were identified in the SCR. Of these, 132 patients had Stage I–III (35%) non-metastatic cancer, and the remaining 241 patients had Stage IV (65%). Although four controls had been matched to each case, 20 had died before their case was assigned a cancer diagnosis, so a total of 1472 controls were generated. The characteristics of the study sample is shown in Table 1. The disease burden for cases and controls was similar regarding the median number of unique diagnostic codes in the year before cancer diagnosis. Data retrieved from the regional database for radiology information (EIA) showed that 151 (40%) out of 373 patients with LC had been referred by a GP for a first chest X-ray in the year prior to cancer diagnosis (Figure 1). Hence, the majority of patients (51%) had been referred for chest X-ray by physicians in secondary care.

**Table 1.** Sample characteristics of patients with lung cancer and controls

Characteristics	Patients with lung cancer, *n* = 373	Controls, *n* = 1472
Median age at diagnosis, years (range)	69 (30–93)	70 (30–93)
Female, *n* (%)	178 (48)	706 (48)
Male, *n* (%)	195 (52)	766 (52)
Age <60 years, *n* (%)	61 (16)	242 (16)
Age 60–80 years, *n* (%)	264 (71)	1046 (71)
Age >80 years, *n* (%)	48 (13)	184 (13)
Stage I–III (M0^a^), *n* (%)	132 (35)	
Stage IV (M1^a^), *n* (%)	241 (65)	
Median number of consultations per patient in year before cancer diagnosis, *n* (IQR)	5 (3–9)	4 (2–7)
Median number of unique diagnostic codes per patient in year before cancer diagnosis, *n* (IQR)	6 (4–10)	6 (3–9)

^a^TNM Classification of Malignant Tumours code. IQR = interquartile range.

### Variables

After the univariable conditional regression was done, there were 15 significant variables left (*P*<0.05) for patients with non-metastatic cancer and 23 for patients with metastatic cancer. The variables with an odds ratio of >1.5 are presented in Table 2. After multivariate conditional regression, several significant variables were found to be independently associated with nMLC, but because there were too few cases for each combination of features, no calculation of positive predictive values could be performed. Even though all the patients included in this study consulted a GP in the year prior to their LC diagnosis, there were differences in their diagnostic profile depending on whether they had been referred for their first chest X-ray by their GP or from secondary care (Table 3). In total, 40% of the patients referred for their first chest X-ray from primary care had nMLC, compared to 30% of those referred from secondary care. The clinical features were 2.7 times more frequent (337 versus 126) in referral letters for chest X-ray than the corresponding features in the healthcare database (Table 4).

**Table 2.** Univariable analysis of diagnoses depending on stage with odds ratio >1.5^a^ in patients in primary care during 12 months before lung cancer diagnosis

Stage I–III (M0^b^)			Stage IV (M1^b^)		
**ICD-10 code and diagnosis**	**Prevalence %**	**OR (95% CI)^c^**	**ICD-10 code and diagnosis**	**Prevalence %**	**OR (95% CI)^c^**
D51 Vitamin B12 deficiency anaemia	3.8	6.7 (1.6 to 27.9)	L20 Atopic dermatitis	1.2	12.0 (1.2 to 115.4)
R060 Dyspnoea	8.4	5.0 (2.0 to 12.7)	R042 Haemoptysis	2.1	9.6 (1.9 to 49.7)
J42 Unspecified chronic bronchitis	3.8	5.0 (1.3 to 18.6)	I26 Pulmonary embolism	1.7	8.0 (1.5 to 43.7)
J44 COPD	20.6	4.3 (2.4 to 7.5)	W00 Fall due to ice and snow	1.7	8.0 (1.5 to 43.7)
I73 Other peripheral vascular diseases	4.6	4.2 (1.3 to 13.9)	M05 Rheumatoid arthritis with rheumatoid factor	2.9	4.7 (1.6 to 13.9)
B34 Viral infection of unspecified site	5.4	4.0 (1.4 to 11.4)	N20 Calculus of kidney and ureter	2.1	4.5 (1.2 to 17.1)
R05 Cough	13.8	3.8 (2.0 to 7.5)	W19 Unspecified fall	2.5	4.3 (1.3 to 14.3)
J18 Pneumonia	12.2	3.2 (1.6 to 6.2)	J18 Pneumonia	9.5	3.8 (2.1 to 7.0)
R52 Pain, unspecified	10.7	2.3 (1.1 to 4.7)	G40 Epilepsy and current seizures	2.5	3.7 (1.2 to 11.6)
N30 Cystitis	14.5	2.0 (1.1 to 3.6)	R05 Cough	14.9	3.6 (2.2 to 5.8)
J20 Acute bronchitis	16.0	1.8 (1.1 to to 3.2)	I73 Other peripheral vascular diseases	7.0	3.6 (1.8 to 6.8)
M54 Back pain	18.3	1.8 (1.1 to 3.1)	R22 Localised swelling, mass, and lump of skin and subcutaneous tissue	3.7	3.1 (1.3 to 7.5)
			M06 Other rheumatoid arthritis	2.5	3.0 (1.0 to 8.6)
			J44 COPD	15.3	3.0 (1.9 to 4.7)
			R060 Dyspnoea	5.8	2.5 (1.3 to 4.9)
			K51 Diverticular disease of intestine	3.7	2.4 (1.0 to 5.4)
			J20 Acute bronchitis	14.9	2.3 (1.5 to 3.5)
			R52 Pain, unspecified	11.2	2.0 (1.3 to 3.3)
			M54 Back pain	19.8	2.0 (1.3 to 2.8)

^a^Odds ratio are calculated between cases and controls. Diagnostic codes with OR <1.5 are omitted. ^b^TNM Classification of Malignant Tumours code. ^c^ *P*<0.05. COPD = chronic obstructive pulmonary disease.

**Table 3.** Univariate analysis of diagnoses referred from primary or secondary care to the first chest X-ray during 12 months before lung cancer diagnosis^a^

Primary care chest imaging referral (*n* = 151)			Secondary care chest imaging referral (*n* = 190)		
**ICD-10 code and diagnosis**	**Prevalence, %**	**OR (95%CI)^b^**	**ICD-10 code and diagnosis**	**Prevalence, %**	**OR (95%CI)^b^**
J42 Chronic bronchitis	5.3	14.9 (3.1 to 70.4)	R01 Cardiac murmurs and other cardiac sounds	1.6	12.0 (1.2 to 115.4)
R042 Haemoptysis	3.3	10.0 (1.9 to 51.5)	Z51 Encounter for other aftercare and medical care	2.1	8.0 (1.5 to 43.7)
W19 Unspecified fall	2.6	8.0 (1.5 to 43.7)	I73 Other peripheral vascular diseases	8.0	5.4 (2.5 to 11.8)
L20 Atopic dermatitis	2.6	8.0 (1.5 to 43.7)	N20 Calculus of kidney and ureter	2.1	4.7 (1.0 to 21.4)
R05 Cough	22.5	7.2 (4.0 to 13.0)	M06 Rheumatoid arthritis, unspecified	4.3	4.6 (1.7 to 12.6)
J18 Pneumonia	15.9	5.4 (2.9 to 10.0)	J44 COPD	17.2	3.8 (2.2 to 6.3)
R700 Elevated erythrocyte sedimentation rate	2.6	5.3 (1.2 to 23.8)	F17 Mental and behavioural disorders due to use of tobacco	6.4	3.7 (1.7 to 8.3)
R49 Voice and resonance disorders	3.3	4.8 (1.3 to 18.1)	M05 Rheumatoid arthritis with rheumatoid factor	3.2	3.4 (1.2 to 10.2)
Z72 Problems related to lifestyle	4.6	4.6 (1.5 to 13.6)	Z13 Special screening examination for other diseases	3.7	3.7 (1.3 to 10.8)
R060 Dyspnoea	7.3	3.8 (1.6 to 8.8)	R060 Dyspnoea	5.3	2.8 (1.2 to 6.4)
R52 Pain, unspecified	14.6	3.6 (2.0 to 6.6)	M54 Dorsalgia	22.3	2.3 (1.5 to 3.5)
B34 Viral infection	4.6	3.5 (1.3 to 9.6)	J18 Pneumonia	6.9	2.2 (1.1 to 4.5)
J44 COPD	18.5	3.4 (2.0 to 5.7)	I25 Chronic ischaemic heart disease	14.9	1.6 (1.0 to 2.7)
J20 Acute bronchitis	19.9	3.2 (1.9 to 5.4)			
G47 Sleep disorders	4.0	3.0 (1.0 to 8.6)			
I80 Phlebitis and thrombophlebitis	4.6	2.8 (1.0 to 7.2)			
M53 Dorsopathy	5.3	2.6 (1.0 to 6.6)			

^a^Odds ratio calculated between cases and controls. Diagnostic codes with OR <1.5 are omitted. ^b^ *P*<0.05. COPD = chronic obstructive pulmonary disease.

**Table 4.** ICPC-2 codes^a^ in letters of referral for chest X-ray compared with corresponding ICD-10 codes in the regional healthcare database

ICPC-2 codes in letters of referral for chest X-ray	Codes recorded, n	Proportion of total ICPC-2 codes, %	ICD-10 codes in the regional healthcare database	Codes recorded, *n*	Proportion of total ICD-10 codes, %
A23 Risk factor NOS	70	17.5	F17 Mental and behavioural disorders due to use of tobacco Z72 Problems related to lifestyle	13	1.4
R05 Cough	65	16.3	R05 Cough	34	3.7
A91 Abnormal result investigation NOS	49	12.3	R79 Other abnormal findings of blood chemistryR919 Abnormal findings on diagnostic imaging of lung	2	0.2
R02 Shortness of breath/dyspnoea	42	10.5	R060 Dyspnoea	9	1.0
R95 COPD	19	4.8	J44 COPD	28	3.0
A04 Weakness/tiredness	17	4.3	R53 Tiredness	7	0.8
T08 Weight loss	15	3.8	R63 Symptoms and signs concerning food and fluid intake	0	0.0
R01 Pain respiratory system	13	3.3	R07 Pain in throat and chest	6	0.6
R24 Haemoptysis	12	3.0	R042 Haemoptysis	5	0.5
L04 Chest symptom/complaint	11	2.8	R07 Pain in throat and chest(included in the results of ICPC-2 code R01)		
R25 Sputum/phlegm abnormal	9	2.3	R09 Other symptoms and signs involving the circulatory and respiratory system	0	0.0
R03 Wheezing	8	2.0	R060 Dyspnoea(included in the results of ICPC-2 code R01)		
R81 Pneumonia	7	1.8	J18 Pneumonia	22	2.4
**Total**	337	84.7	**Total**	126	13.6

^a^Occurring in >1% of clinical features. COPD = chronic obstructive pulmonary disease. NOS = Not otherwise specified.

## Discussion

### Summary

The study identified 12 features that were associated with nMLC, of which eight were also in common with metastatic LC. The features with the highest OR for nMLC were vitamin B12 deficiency anaemia, dyspnoea, and chronic bronchitis. Clinical features that were GPs’ reasons for request for chest X-ray were almost three times more frequent in referral letters compared to the corresponding diagnostic codes in the medical records.

### Strengths and limitations

The main strength of this study is that it is total population-based. All patients with cancer were identified through the SCR, so there is no selection bias and the completeness of the register is very high.^16^ The study looked at the clinical features presented during the year before LC diagnosis, because knowing these has major consequences for timelier and earlier LC diagnosis, which in turn affects prognosis. The use of diagnostic codes is another strength of the study. However, this could also be considered a limitation because not all the symptoms for which patients consulted a GP would be recorded as a diagnostic code in their medical record, as other fields of research in primary care databases have shown.^25^

Most cancer symptoms occur 3–6 months before the cancer diagnosis, but a longer time than the one used in this study may be needed for observation.^26^ The lack of laboratory results to validate the diagnoses of vitamin B12 deficiency anaemia, which had the strongest association with nMLC is another limitation. The absence of smoking status of patients with cancer is a limitation too, as the symptomatology of smokers has more severe implications than that of non-smokers.^27^

Another limitation is that the authors were unable to design a risk assessment tool for nMLC in primary care. This is due either to the lack of a large enough sample, resulting in the inability to capture combinations of features, or to a truly low frequency of combination of features in the non-metastatic population, which may not be detected even with a larger sample size.

The low prevalence of clinical features in the regional healthcare database in comparison to clinical features in referral letters for X-ray is probably due to the former consisting mainly of diseases and the latter of symptoms. Another explanation could be that the reimbursement system for primary care providers is partly based on the disease burden of the patients, which favours disease codes over symptom codes.

### Comparison with existing literature

To the authors' knowledge, this is the first study to present the clinical features of LC in patients with a non-metastatic disease. This is also the first study to present vitamin B12 deficiency anaemia as being a risk marker for nMLC. Perhaps this finding is a paraneoplastic phenomenon. However, previously published studies have shown that individuals with vitamin B12 deficiency anaemia are at increased risk for other cancers, such as gastric cancers and blood malignancies.^28,29^ A recent systematic review from the UK has suggested that patients with thrombocytosis in primary care have an increased risk of several cancers, among them LC, which this study was unable to show as it lacked data on blood test results.^30^ Another UK study from primary care has reported association with LC in the first year after presentation with back problems, which is in line with this study's findings.^31^ In this study, the clinical information in referral letters for chest X-ray was extensive, in contrast to what has been reported in the literature.^32^

A Danish study showed that patients with LC and chronic obstructive pulmonary disease (COPD) had more contacts in primary care in the 11 months prior to diagnosis than did patients with LC but without COPD. Thus, having COPD can mask symptoms of LC.^33^ This is in line with the findings presented here, that COPD is a risk marker in patients with both non-metastatic and metastatic LC.

An LC assessment tool for primary care has been developed and implemented in the UK.^27,34^ As this study did not result in a scoring instrument for LC, the results presented here are not easily comparable. The UK assessment tool makes no distinction between features depending on tumour stages. Compared to the UK study that found nine clinical features associated with LC, the present authors found only two in common with the nMLC group: dyspnoea and cough. In this study, haemoptysis was only associated with metastatic LC.

QCancer® is another risk prediction algorithm.^35,36^ It is designed to estimate the 10-year risk of having 11 common cancers, including LC, and is based on both symptoms and risk factors. The symptoms studied were mostly 'red flag' symptoms and risk factors associated with LC. The tumour stages at diagnosis were not recorded, and there was no evidence as to whether use of the tool was likely to lead to identification of LC at an earlier stage. The clinical features presented in this study are the result of all symptoms and diseases being registered as diagnostic codes in general practice, and not just features that have been reported to be associated with LC in other studies.

A large UK study has developed and validated a risk prediction model for LC, using a combination of patients’ sociodemographic and early clinical features identified 4–12 months before diagnosis.^37^ Again, the study was hard to compare with this one, as the clinical features were not associated with different tumour stages. The symptoms cough, dyspnoea, chest infections, and lower respiratory tract infections had similar OR as in this study, while haemoptysis had an OR twice as large compared to that in the present study's findings.

A recently published systematic review of risk prediction tools for patients with LC based on UK primary care data compared five different tools.^38^ There was not sufficient evidence to recommend any of them because of the lack of external validation, evaluation in clinical practice, and cost impact. Also, none of the tools differentiated between symptoms depending on tumour stage.

Existing risk prediction tools are not designed for identification of early-stage LC. However, LC screening of high-risk target groups with LDCT has shown many promising results in the detection rate of early-stage LC. This screening has been implemented in the US, but the results have been discouraging so far, because <4% of the eligible 6.8 million smokers in the US have received LDCT screening.^39^

### Implications for research

Patients with nMLC cannot be easily identified by symptoms. However, this study showed that referrals for chest X-ray from primary care resulted in a detection rate of 40% of patients with nMLC.
